# Supplementary material for: Saccharomyces boulardii (CNCM I-745) improves intestinal damage in sepsis by remodeling intestinal flora structure
Source: Front Cell Infect Microbiol. 2025 Jul 31;15:1602792. doi: 10.3389/fcimb.2025.1602792 (PMC12350329; doi:10.3389/fcimb.2025.1602792)
Supplement: Supplementary file 1 [file DataSheet1.zip › summary/summary/7_Differential_analysis/1_diff_abundance/6_Genus/Group.CW0_vs_AW0/Group.CW0_vs_AW0_diff_barplot.pdf]

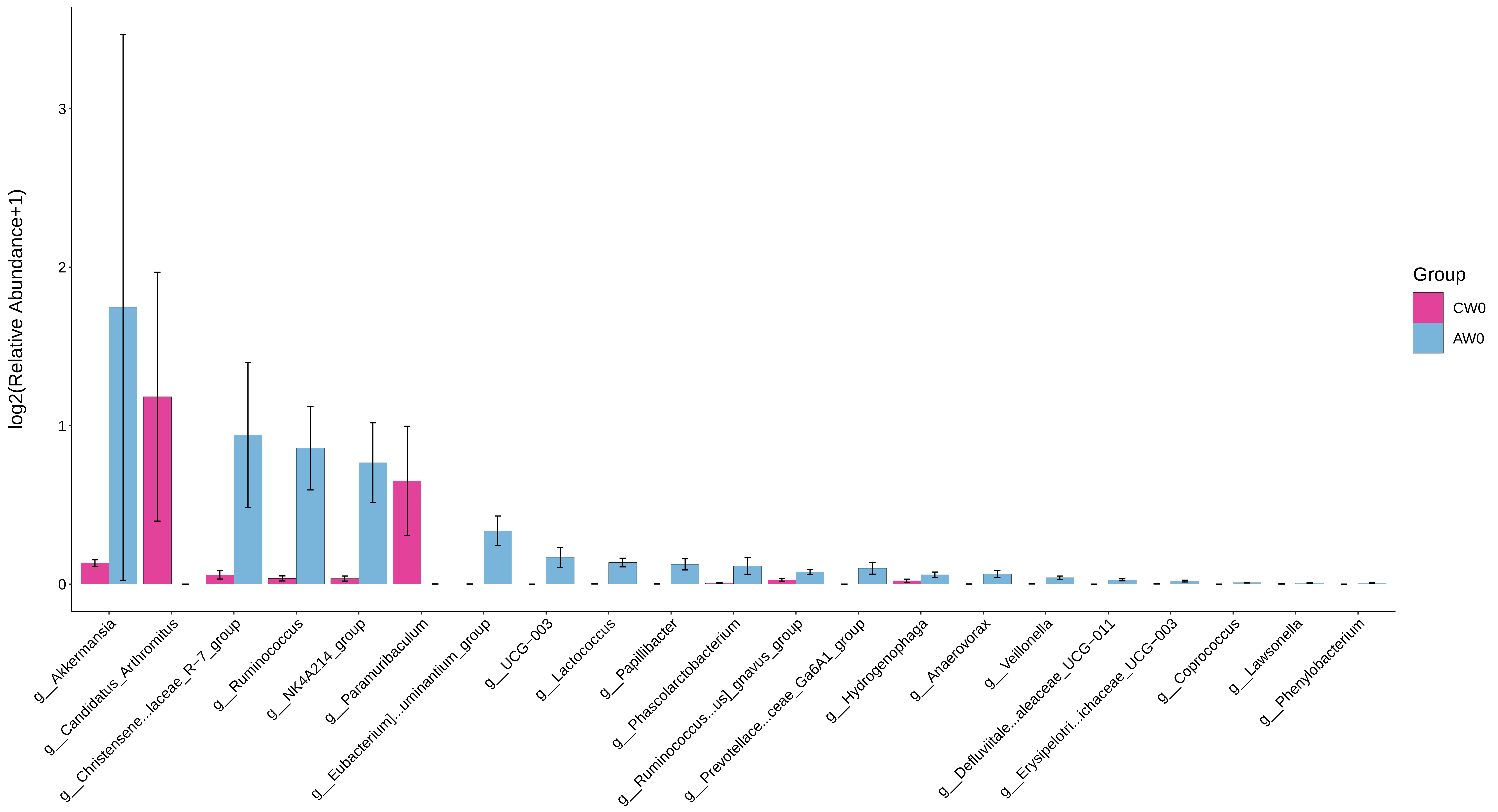

log2(Relative Abundance+1)
0
1
2
3
g__Akkermansia
g__Candidatus_Arthromitus
g__Christensene...laceae_R−7_group
g__Ruminococcus
g__NK4A214_group
g__Paramuribaculum
g__Eubacterium]...uminantium_group
g__UCG−003
g__Lactococcus
g__Papillibacter
g__Phascolarctobacterium
g__Ruminococcus...us]_gnavus_group
g__Prevotellace...ceae_Ga6A1_group
g__Hydrogenophaga
g__Anaerovorax
g__Veillonella
g__Defluviitale...aleaceae_UCG−011
g__Erysipelotri...ichaceae_UCG−003
g__Coprococcus
g__Lawsonella
g__Phenylobacterium
Group
CW0
AW0
